# Supplementary material for: The impact of dual- versus single-dosing and fatty food co-administration on albendazole efficacy against hookworm among children in Mayuge district, Uganda: Results from a 2x2 factorial randomised controlled trial
Source: PLoS Negl Trop Dis. 2023 Jul 3;17(7):e0011439. doi: 10.1371/journal.pntd.0011439 (PMC10317238; doi:10.1371/journal.pntd.0011439)
Supplement: S1 Protocol — (DOCX) [file pntd.0011439.s002.docx]

Cross-sectional prevalence study of schistosomiasis and soil-transmitted helminthiasis with nested open-label randomised controlled study of evaluating the impact of fatty meal co-administration and double-dosing on albendazole effectiveness against hookworm infection among school-aged children in Mayuge district: Implications for Mayuge NTDs Elimination (MANE) Project

(2019-2021/ NTD/ World Vision)

June 2021

Table of Contents

[1. Background 2](#_Toc120738485)

[1.1 Introduction 2](#_Toc120738486)

[2. Aims and objectives 6](#_Toc120738487)

[2.1 General objective 6](#_Toc120738488)

[2.2 Specific objective 6](#_Toc120738489)

[3. Methodology 6](#_Toc120738490)

[3.1 Study area 7](#_Toc120738491)

[3.2 Study design, inclusion and exclusion criteria 7](#_Toc120738492)

[3.3 Sampling method for prevalence survey 9](#_Toc120738493)

[3.4 Randomisation into four groups for the trial 9](#_Toc120738494)

[3.5 Data collection 10](#_Toc120738495)

[3.6 Sample size and sampling method 12](#_Toc120738496)

[3.7 Data management and analysis 13](#_Toc120738497)

[4. Dissemination Plan 14](#_Toc120738498)

[5. Data ownership 14](#_Toc120738499)

[6. Timeline 15](#_Toc120738500)

[Reference 16](#_Toc120738501)

1. Background

## Introduction

Soil-transmitted helminths (STH) infection, also known as intestinal worm infection, is a very common condition but remains a neglected condition globally. There are three dominant STH: roundworm (Ascaris lumbricoides), whipworm (Trichuris trichiura) and hookworm (Ancylostoma duodenale and Necator americanus)(1,2). Hookworm is the main STH causing anaemia, contributing to approximately 4 million DALYs globally per year (3).  STH primarily affects the world’s deprived populations, causes significant health and socio-economic repercussions and constitutes an important public health problem in developing countries. The WHO estimates that STH affects more than 1.5 billion people worldwide(4). Globally, the prevalence of hookworm and *Ascaris lumbricoides* is estimated as 13.6% for both species, while the prevalence of *Trichuris trichiura* is estimated as 11.6% in sub-Saharan Africa (4).

Schistosomiasis is a chronic waterborne diseases caused by blood flukes of the genus Schistosoma. It affects approximately 240 million individuals in the tropical and subtropical areas, and is prevalent in more than 78 countries, with nearly 800 million people exposed to the risk of infection(5,6). Schistosomiasis is a major cause of morbidity and mortality in Africa, South America, the Caribbean, the Middle East and Asia(7).  *Schistosoma mansoni* causes intestinal schistosomiasis producing severe organ pathologies such as hepatosplenomegaly with periportal liver fibrosis and portal hypertension which progress from abdominal pain and bloody diarrhoea. Urogenital schistosomiasis caused by *Schistosoma haematobium* leads to haematuria, dysuria, hydronephrosis, female genital schistosomiasis and calcification of the bladder.

Preventive chemotherapy has been a backbone strategy to control STH and schistosomiasis, but for effectively controlling these NTDs, improved access to safe water, adequate sanitation, vector control, and health education is also needed and can accelerate progress towards elimination of these NTDs(8–10).

The WHO recommends benzimidazole drug (400mg albendazole or 500mg of mebendazole) against STHs, with pre-school-aged children (one to four years old) and school-aged children(five to 14 years old) living in the area where the prevalence of STH is over 20% targeted for annual or biannual treatment (11). For schistosomiasis, preventive chemotherapy with praziquantel is targeted at school-aged children, as they harbour the heaviest worm burden in a population. High-risk groups, such as fishermen and women who frequently visit contaminated water sources, are also targeted for mass treatment(12).

The WHO target is for at least 75% children to be covered through mass drug administration in areas at risk for STHs and schistosomiasis. Based on the WHO guidelines for evaluating STH and schistosomiasis at the community level(13), 9-10 years old school children should be the primary target population (i.e. Primary 4 and 5 students in the Ugandan education system)

The Uganda NTD Master plan (2017-2022), highlights the high burden of the diseases, particularly in poor communities with limited access to health care, and inadequate information and means of prevention and control measures. The plan further categorises NTDs into two groups; those amenable to preventive chemotherapy (PC-NTDs) and those controlled through case management (CM-NTDs). STHs and schistosomiasis are targets for preventive chemotherapy. Furthermore, the Uganda government was a signatory to the international treaties and conventions to eliminate targeted diseases and is committed to controlling and eliminating targeted NTDs by the year 2020. But, now, the WHO 2030 NTD roadmap suggests new global targets to prevent, control, eliminate, and eradicate a set of 20 NTDs. The WHO 2030 roadmap emphasises the necessity of shifting the roadmap from previous approaches to attain the goal of 2030: 1) Accelerating programmatic action within integrated platforms for delivery of intervention, 2) Cross-cutting approaches must replace the single disease elimination approach, 3) Country ownership is essential to sustain the achievement obtained through previous NTDs programs(14). The Ugandan government launched its national sustainable plan for NTDs in February 2021 as one of the first NTD endemic countries to announce a comprehensive NTD sustainability plan(15). In this plan, the Ugandan government emphasises:

1) Multi-sectoral collaboration.

2) Integrating NTD services within the broader health care delivery system.

3) Strengthening advocacy to increase the portion of domestic funding for NTD from 12% to 25% by 2025.

4) Identification indicators for monitoring the progress on NTD programs.

- - 1. **Mayuge NTDs Elimination (MANE) project**

World Vision, funded by Korea International Cooperation Agency (KOICA), has implemented comprehensive NTD elimination programs targeting STHs and schistosomiasis in Mayuge district since 2019, known as the Mayuge NTDs Elimination (MANE) project. The MANE project is a three-year program composed mainly of mass drug administration (MDA), water, sanitation and hygiene (WASH), and awareness increasing programs for the communities and health workers. For the WASH component, of 13 sub-counties of Mayuge district, two sub-counties covering 25,000 population will benefit from the piped water system. The MANE project has been constructing constructs nineteen public latrines in selected schools and implemented community-led total sanitation (CLTS) programs in 25 villages. For increasing awareness program, the MANE project has utilised a citizen voice and action (CVA) program developed by World Vision to improve people’s awareness of their public health issues and strengthen the relationship between the community and the authorities.

In 2019, a prevalence survey was done as the baseline survey to understand the burden of STH and schistosomiasis in Mayuge district. According to the WHO guideline, the MANE team selected the samples from 1,123 9-10 years old children who are equivalent to P4 and P5 grade students in the Ugandan education system (13). Baseline prevalence of schistosomiasis was 27.2% (305/1,123) and prevalence of STH was 15.8% (177/1,123). Among the 177 identified STHs positive samples, 96% (n=170) of STHs were hookworms.

- - 1. **Hookworm dominance in the prevalence of STH**

Mayuge district has implemented an annual MDA program against STHs targeting school-aged children since 2003. According to the MANE baseline survey of 1,123 children in the same age group in July 2019, the prevalence of hookworm was 15.1%, while the prevalence of Ascaris lumbricoides was 0% and prevalence of Trichuris trichiura was 0.6%. Thus, in Mayuge district, hookworm is the most common STH, and will be the focus of the current study. Considering the cure rate for hookworm by single-dose albendazole is thought to be 78.4%(16), and the high program coverage of MDA for STHs (77.2%, 81.4%, and 75% in 2012, 2014, and 2016, respectively), the continued burden of hookworm in Mayuge district suggests that hookworm has developed mechanisms to overcome the MDA campaigns.

- - 1. **Possible mechanisms for continued high prevalence of hookworm infection**

Proposed theories include the possibility that annual single-dose albendazole use for MDA is not enough to effectively treat hookworm; organisms have developed albendazole resistance traits; or that the effect of annual monotherapy with benzimidazole drugs has been handicapped by intrinsic environmental and social factors(17).

- - - 1. **Drug resistance in hookworm**

It has been demonstrated that overuse of benzimidazole drugs in animals is associated with the development of anthelmintic resistance, such as single nucleotide polymorphisms (SNPs) in the beta-tubulin isotype 1 gene at codons 167, 198 and 200 (18,19). In the same vein, benzimidazole has regularly been used to control STHs that may cause drug resistance against STHs in human(17). However, in hookworm-infected humans, benzimidazole drug resistance has not been reported yet(20,21). It appears more plausible that the current dose of albendazole targeting hookworm infection is inadequate to effectively control hookworm infection in humans due to reasons other than albendazole resistance.

- - - 1. **Infection intensity**

The cure rate of anthelminthic drugs against STH decreases when faecal egg count is high at pre-treatment (22). But, it is also known that the fecundity of residual female hookworm increases after treatment, caused by the relaxation of adult hookworm density(23,24). It means that the faecal egg count reduction test (FECRT) can reveal a wide range of drug efficacy against STHs within different human STHs cases. Kotze and Kopp suggested exclusion of cases with the heaviest infections from a FECRT to improve the interpretation of FECRT as an indicator of drug efficacy(24).

- - - 1. **Lack of absorbing albendazole and increasing albendazole absorption with fatty meal**

When it comes to albendazole absorption in the intestine, research has shown that albendazole absorption increases five to six-fold if taken with a fatty meal (25,26). Ceballo *et al*. assessed the serum pharmacokinetics of albendazole and its metabolites in eight Argentine adults. They showed a high variation of serum concentration of albendazole sulphoxide (ABZSX) among participants, which can be one reason why people show different cure rates when taking the same dose of albendazole (27). Therefore, it is necessary to consider and evaluate approaches for increasing albendazole absorption in order to improve drug efficacy against human hookworm, since individuals have different albendazole absorption.

In previous studies done in a range of countries; Brazil, Cameroon, Cambodia, China, Ethiopia, India, Lao PDR, Tanzania, and Vietnam, the cure rate of single-dose albendazole for hookworm ranged from 36 to 93%(22,28–30). According to the study done in Gabon by Adegnika *et al*., the cure rate of double-dose albendazole for hookworm was 92%; on the other hands, the cure rate of single-dose albendazole was 54% (31).

Avocado is one of the easily accessible foods containing high fat in rural areas of Uganda. 100 g of avocado contains 15g of fat, 2g of protein, and 8.5g of carbohydrate (32).

- - - 1. **Albendazole dissolution**

Albonico *et al* (2007)’s Nepal study investigated the efficacy of single albendazole manufactured in different pharmaceutical companies including the original manufacturer (GSK) and two other generic drugs targeting STHs(33). Dissolution time among the three tested drugs differed. The two generic drugs did not dissolve as well as the original drug made by GSK, which affected the cure rate of albendazole against hookworm. For hookworm, with albendazole made by GSK the cure rate was 74.3% compared to, with two other generic drugs, 53.3% and 50.7% respectively. Lower dissolution affects lower active doses for anthelminthic effect in gastrointestinal tract. Therefore, children must be informed to chew the chewable albendazole before swallowing to maximise drug efficacy against hookworm. Belew *et al* (2015)’s Ethiopia study also shows different cure rate between two albendazole brands against hookworm(34). One drug is more efficacious than the other drug with significant difference (*p*-value < 0.05) of egg reduction rate (98.1% versus 88.7%). The authors explained that low albendazole dissolution rate reduced bioavailability, which eventually lowered in-vivo drug efficacy against hookworm. They demonstrated that one brand of albendazole’s dissolution rate was four times higher than the other brand of albendazole used in the study.

- - - 1. **Sociocultural and environmental factors**

In addition, sociocultural and environmental factors affecting the high incidence rate of hookworm infection have been reported in multiple study populations (35–39)(40,41), which would be plausible reasons for hookworm reinfection among treated people. These factors require further investigation in Uganda.

| **Risk factors** | **Hookworm infection** | **Country** | **Reference** |
| --- | --- | --- | --- |
| Boys | OR=2.33 (95% CI=1.23-4.42, *p*-value:0.01) | Honduras | Gabrie *et al*. |
| Population density | OR=0.86 (95% CI=0.63-0.73, *p*-value:0.001) | Tanzania | Riess *et al*. |
| Temperature | Correlation with incidence of hookworm:  *r*_s_ = - 0.468 (p-value: 0.018) | Sri Lanka | Gunawardena *et al*. |
|  | Mean annual land surface temperature  *day: OR=0.81(95% CI=0.75-0.88, *p*-value:0.001)  *night: OR=154(95% CI=1.44-1.64, *p*-value:0.001) | Tanzania | Riess *et al*. |
| Barefoot | Incidence rate ratio(IRR)= 4.2 (95% CI= 1.2-14.5, *p*-value: 0.023) | Thailand | Jirannankul *et al*. |
|  |  |  |  |
| Raising animals | IRR=4.8 (95% CI=1.9-11.8, *p*-value : 0.001) – raising buffalos | Thailand | Jirannankul *et al*. |
| Water exposure |  | | |
| Wastewater farmers | OR=31.4 (95% CI=4.1-243, *p*-value: 0.001) compared to regular farmers on risk of nematode and hookworm infection | Pakistan | Ensink *et al*. |
| Bathing and washing with water from water fall | OR=2.33 (95% CI=1.35-4.01, *p*-value : 0.001) | Sri Lanka | Gunawardena *et al*. |
| The use of wells | OR=2.35 (95% CI=1.29-4.30, *p*-value : 0.005) | Sri Lanka | Gunawardena *et al*. |
| Lack of latrines | OR=1.60 (95% CI=1.01-2.53, *p*-value : 0.045) | Sri Lanka | Gunawardena *et al*. |
|  | OR=1.02(95% CI=1.01-1.04, *p*-value : 0.002) | Tanzania | Riess *et al*. |
|  | OR=1.90 | Kenya | Olsen *et al*. |
| School deworming(None or only once a year) | OR=10.57 (95% CI=4.53-24.66, *p*-value:0.001) | Honduras | Gabrie *et al*. |

(Table 1) Risk factors for hookworm infection

1. Aims and objectives

***Aims*:** After identifying the prevalence of STHs and schistosomiasis in Mayuge district to evaluate the impact of the MANE project on the burden of STHs and schistosomiasis, the study aims to assess the efficacy of albendazole with different doses and when co-administrated with fatty meals against hookworm, the dominant form of STHs in Mayuge district, Uganda. This study is designed to verify why hookworm is still prevalent regardless of more than fifteen years’ MDA program in Mayuge district and suggest an alternative MDA option for reducing hookworm prevalence. The evidence generated from this study will guide effective MDA option for STHs.

## 2.1 General objective

The general objective is to investigate approaches for improving the effectiveness of albendazole treatment for curing hookworm infection.

## 2.2 Specific objective

i. To determine the effect of albendazole administration with a fatty meal such as avocado, versus albendazole administration without a fatty meal, on hookworm cure rate and egg reduction rate.

ii. To determine the effectiveness of dual-dose (400mg/day, two consecutive days) versus single-dose (400mg) albendazole treatment regimens on hookworm cure rate and egg reduction rate.

iii. To identify and evaluate environmental, social and cultural variables affecting hookworm infection, and cure rate and egg reduction rate of albendazole against hookworm.

1. Methodology

This study will be embedded within the framework of the MANE project. The baseline prevalence survey was completed in July 2019, and the second prevalence survey is scheduled in September 2021 to evaluate the impact of programs implemented between 2019 and 2021.

## 3.1 Study area

Mayuge district is located in south-eastern Uganda and borders on Jinja, Iganga, Namayingo, and Bugiri. It is situated next to Lake Victoria. Mayuge is 146 km away from Uganda’s capital, Kampala. It has 13 sub-counties. According to the result of the registration activity in 2020, which was done as a part of the MANE project, Mayuge’s population is 509,118; with 95,418 children under five; and 156,182 children between five and 14 years old. It has a total of 504 primary schools, 41 health centres (HCs), and one hospital (HC II-34, HC III-5, HC IV-2). There are 1,536 health workers, and they work to raise awareness of MDA.

## 3.2 Study design, inclusion and exclusion criteria

The study will have two phases. The first phase will be a descriptive cross-sectional study which will evaluate the prevalence of STHs and schistosomiasis in Mayuge district as the follow-up survey of the MANE project.

In the second phase, children who have hookworm infection in this cross-sectional survey, will then take part in a randomised controlled trial with factorial design to assess impact of dual- vs single-dose albendazole and impact of taking albendazole with fatty foods on cure rate and egg reduction rate. Outcomes will be assessed at a follow-up visit 21 days after albendazole treatment.

In Mayuge district, primary four and primary five students will be the target population. Target schools will be selected and a given number of the sample from each selected school confirmed. Children whose parents/guardian signed consents for the children participating in the prevalence survey, and in the nested trial if hookworm infection is detected, will be included.

**Inclusion criteria are**

1. Children attending randomly selected primary 4 and 5 classes in one of the randomly selected schools and signed for child assent form
2. Children whose parents/guardian provide written informed consent for the prevalence survey and for the nested trial if hookworm infection is detected.

**Exclusion criteria are:**

1. Children who received praziquantel and albendazole within three months before the prevalence survey to avoid the residual effect of previously administrated drugs of praziquantel and albendazole.
2. Children who have a history of allergic reaction to either albendazole or praziquantel.
3. Children whose stool results come out as *Ascaris lubmricoides, Trichuris trichiura* ^[[1]](#footnote-1)^and other parasites infection apart from hookworm infection will not take part in the trial.

**Fig 1: Study enrolment, randomisation and evaluation plan**

**PHASE I STUDY**

**1,650 school-aged children will provide stool samples**

**Questionnaire survey will be done together**

**PHASE II STUDY**

**212 positive cases of hookworms assuming prevalence of hookworm is 12%**

**212 positive cases of hookworms will provide stool samples**

**Faecal egg counts from hookworm positive**

**stool samples**

**Albendazole**

**Dual-dose treatment with avocado (n=53)**

**Albendazole**

**Dual-dose without avocado treatment (n=53)**

**Albendazole**

**Single-dose treatment with avocado**

**(n=53)**

**Albendazole**

**Single-dose treatment without avocado (n=53)**

**21 days later after treatment**

**(5% follow up loss)**

**Cure rate and egg reduction rate will evaluated**

## 3.3 Sampling method for prevalence survey

**3.3.1 Selection of schools**

Of 504 schools in Mayuge district, 33 schools will be selected randomly and independently from the school list stratified by the parish and sub-county using probability proportional to size selection (PPS). Before the sample selection, the sampling frame will be sorted within each sampling stratum first, by size. The required number of schools shall then be selected by probability proportional to size (PPS).

**3.3.2 Selection of classes**

Within each selected school, a class (40-50 children) will be selected among the P4 and P5 classes (9-10-year-old schoolchildren) using a table of random numbers or a lottery method. If only one class of this age group is present that class has to be selected. If the number of children present in the selected class is lower than 35, a second class will be selected and all children in both classes examined.

## 3.4 Randomisation into four groups for the trial

When positive hookworm cases are identified through the prevalence survey, field workers will notify the students of "positive" results. These students will be requested to visit the school where they attend for participating in the trial. The field researchers will be formed by health personnel from the most accessible health centre IV or hospital-level health facility in Mayuge district: 1) Mayuge health centre IV located in Mayuge town council, 2) Kigandalo health centre IV located in Kigandalo sub-county, 3) Kityerera health centre IV located in Kityerera sub-county, and 4) Buluuba hospital in baitambogwe sub-county. The participants will be instructed to have a meal on the morning of the visit, following the protocol for taking fatty meal with albendazole as outlined in the study of Nagy *et al.*(25). These participants will be randomised into one group among four treatment options by applying the block randomisation method, stratified by health facility. The randomisation block size will be 8, and within each block there will be two participants allocated to each of the four trial arms. A randomisation list will be prepared beforehand using a random number generator. Each health facility will have its own random number list. The participants will take a meal four hours after the drug consumption to exclude the possibility of affecting albendazole absorption by immediate taking foods. But, water, no caffeine drinks, non-alcoholic beverages can be allowed during four hours fasting period.

Albendazole (AGOZOLE 400mg; batch number T11098, produced by AGOG Pharmaceutical Ltd., India) will be used for this trial. This is not a new drug. AGOZOLE albendazole is approved by the National Drug Authority (NDA) of Uganda and is widely available in the Ugandan domestic pharmaceutical market (NDA registered number: NDA/MAL/HDP/3013).

The trial is designed as an open-label randomised controlled trial with four arms:

Intervention group 1: participants will take 400mg of single-dose albendazole with 200 grams of avocado.

Intervention group 2: participants will take dual-dose albendazole without taking avocado. (Participants will take 400mg of single dose albendazole for two consecutive days)

Intervention group 3: participants will take dual-dose albendazole with 200 grams of avocado. (Participants will take 400mg of single dose albendazole with 200 grams of avocado for two consecutive days)

Control group: participants will take 400mg of single-dose albendazole without taking avocado.

For participants in intervention groups 2 and 3, who are taking 400mg of albendazole on two consecutive days, they will be asked to return to the same clinic to do so.

If adverse events occur, the participants who have adverse events will be treated accordingly by the health workers who monitor the participants. If serious adverse events (SAEs) develop, these cases will be reported to VCD after recording the case(s) in the designated form, while managing the SAEs accordingly.

Three weeks after the treatment, the stool collection process will be repeated for those who participate in the treatment. These stool samples will be used to assess cure rate and egg reduction rate following albendazole treatment. If the MANE team find positive hookworm cases on the day 21 of follow up, the MANE team provide him/her alendazole again.

## 3.5 Data collection

Before data collection, the study team will inform the selected school about the study in advance supported by the district education office of Mayuge district.

The role of each researcher will be as below:

1. Enrolment: the MANE team with support by DHO (Dr Eun Seok Kim)
2. Prevalence survey: logistical issues will be led by the MANE team(Dr Eun Seok Kim)
3. Treatment, monitoring AEs, Follow up: the MANE team will organise the work in Mayuge (Dr Eun Seok Kim) with the support/guidance of technical experts of VCD.
4. Overall supervisors: (from the UK) Dr Emily Webb & Dr Kalpana Sabapathy

(from Uganda) Mr. Moses Adriko

1. Organising logistical issues in the field and supervision: Mr. Bomax Otim

**3.5.1 Questionnaire survey**

Trained field workers will administer pre-tested questionnaires to enrolled participants, entering data on tablet PCs. The questionnaire includes sections on: 1) demographic information, 2) general sanitation and hygiene conditions, 3) contact with contaminated water and soil, 4) contact with animals, 5) their habits to use water and life styles.

**3.5.2 Stool sample collections**

Stool samples will be collected from enrolled students from the selected schools by the field workers who will visit the schools, and, as described above, from the students who take part in the albendazole treatment trial three weeks after treatment. The collected stool samples will be contained in a designated stool container secured in a cooling box carried by the field workers. The samples will be transferred to the laboratory and the Kato-Katz examination performed. Delivered stool containers will be secured in a refrigerator before being processed, and contained stools will be utilized for making slides with Kato-Katz method within 24 hours after being delivered to the laboratory.

- - 1. **Organizing laboratory examination**

The WHO recommends the Kato-Katz quantitative method as the standard methods for evaluating prevalence and intensity of soil-transmitted helminthiasis and schistosomiasis in endemic communities. Kigandalo health centre IV will be the base for parasitological examination since this health centre IV has the central laboratory of Mayuge district.

(Collection of stool samples)

Plastic stool containers and applicator sticks are provided in advance

1. Put stool (about 5g) into a plastic container.

2. Take stool to the school.

3. Keep stool in the container in the cooler boxes.

4. The cooler boxes contained stool samples are transported to Kigandolo HC IV for analysis in the afternoon of the same day.

5. Stool samples which are not treated with Kato-Katz method will be contained a refrigerator in the Kigandalo HC IV.

**3.5.4 Parasitological examination for STH and schistosomiasis**

Kato-Katz procedure will be applied to prepare the slide for examination. Six South Korean professors of parasitology who participated in the prevalence survey in 2019 and the health specialist of Mayuge NTD Elimination project, will examine the prepared slides and interpret the slides. The supervisors dispatched from vector borne and NTD control (VCD) division of ministry of health of Uganda will join the prevalence survey of 2021. The supervisors will examine at least 10% of slides which were already examined by the group of professors and the health specialist to ensure the accuracy of the prevalence survey. The results of the parasitological examination will be recorded in the case report form.

Alternatively, if South Korean professors are not permitted to travel due to an international travel ban because of the COVID-19 pandemic, the MANE team would request VCD in advance to form the study team composed of laboratory technicians belonging to Uganda's ministry of health or the Eastern regional authority of Uganda.

## 3.6 Sample size and sampling method

**3.6.1 Sample size**

**For phase I:**

Sample size determination for the prevalence survey will be based on the comparison of prevalence of schistosomiasis and soil-transmitted helminths in Mayuge district in 2019 with the anticipated prevalence of STH and schistosomiasis after two years’ project implementation.

The baseline prevalence of STHs in 2019 was 15.8 % and it is anticipated that MANE program activities will reduce the prevalence of STHs to 12% by the time of the 2021 survey. For schistosomiasis, the prevalence was 27.2% in 2019, and it is estimated that MANE program activities will reduce schistosomiasis prevalence to 18% by the time of the 2021 survey.

Applying to the difference of expecting prevalence of STH in 2021 and proven prevalence of STHs in 2019, a target sample size for the 2021 prevalence survey of 1300 participants would be required for 80% power to detect a difference between 2019 and 2021 STH prevalence at 5% significance level.

**For phase II:**

For the trial phase of the study, the aim is to assess the effect of co-administration with fatty foods, and of dual-dose versus single-dose albendazole on hookworm cure rate. The cure rate of single dose albendazole for hookworms varies between 36% and 93% in previously reported studies. Based on data from Adegnika et al (42), we power our trial to detect a difference in cure rate of 70% versus 50% for both co-administration with fatty foods versus no co-administration, and for dual-dose versus single-dose albendazole.

With type I error probability of 5%, 93 participants in each comparison group will be required for 80% power to detect a difference in cure rate of 70% versus 50%. Therefore, the total target sample size for the trial will be 186. However, allowing for 5% loss to follow-up, i.e. 5% of hookworm positive cases not appearing on the follow up stool test 21 days after treatment, the target sample size will increase to 196. If the expecting prevalence of hookworm in 2021 is 12%, to satisfy the sample size of 196, the target sample size of the prevalence survey of 2021 will be 1,634. Therefore, a total sample size of 1,650 will be sufficient to cover sample size requirements of both the prevalence survey and the nested trial.

Assuming that 50 children will be selected from each school, a total of 33 schools will be randomly selected in order to achieve the required sample size.

**The scenario of not having enough number of participants for the phase II trial:**

If the prevalence of hookworm in the survey is lower than anticipated so that the target sample size of 196 for the phase II trial is not achieved, the MANE team will purposely select hookworm high prevalence school(s) and test for hookworm among those who are not taking part in the phase I prevalence survey. For example, after the phase I prevalence survey, if twenty more participants will be needed, and the prevalence of hookworm in school “A” is 50%, the MANE team will additionally select approximately 40 students in school “A”.

## 3.7 Data management and analysis

Quality control is important to ensure quality outcome of the data. The following measures will be undertaken to secure the quality of this study.

- Completed questionnaires and written notes will be crosschecked immediately after exercise and during the nights of each successive fieldwork to ensure that each relevant question has been asked and the response properly recorded.
- Field workers for stool sample collection and interview will be comprehensively trained before data collection, drawing special attention to interviewing techniques, recording of responses, probing, ethics and data handling/management among others.
- The research instruments will be pre-tested and amended as necessary.
- Appropriate supervision of the entire data collection process will be ensured by the team leader and supervisors.
- Field diaries will be kept by the study team to record any events deemed important for the interpretation of the results.

**3.7.1 Data analysis**

The MANE team will use Open data kit (ODK) of World Vision for data generation, collection and store

1. ODK build: pre-tested questionnaire questions will be transferred into ODK format.
2. ODK collect: pre-trained field workers will collect data from the participants during the prevalence survey
3. ODK aggregate: collected data will be uploaded and stored into World Vision ODK server.

Data saved in the ODK system will be exported into Microsoft Office Excel, and the data will be checked errors, any missing values, and extreme values. The cleaned data will be exported to the statistic program (Stata or R).

Prevalence of STHs and schistosomiasis with 95% confidence intervals will be calculated. The arithmetic eggs count will be obtained from the microscopic examination of the Kato-Katz method applying made slide. A multiplication factor of 24 should be applied to calculate eggs per gram, since the stool quantity in a slide prepared with the Kato-Katz method contains 41.7mg of the stool. The intensity of infection will be categorised by the criteria of WHO guideline as below(13).

|  | Light intensity infections | Moderate intensity infections | Heavy intensity infections |
| --- | --- | --- | --- |
| *A. lumbricoides* | 1-4,999 epg | 5,000-49,999 epg | ≥ 50,000 epg |
| *T.trichiura* | 1-999 epg | 1,000-9,999 epg | ≥ 10,000 epg |
| Hookworm | 1-1,999 epg | 2,000-3,999 epg | ≥ 4,000 epg |
| *S.mansoni* | 1-99 epg | 100-399 epg | ≥ 400 epg |

(Table 2) The threshold of infection intensity by each STH and *S.mansoni*

I will analyse the relationship between the prevalence and the risk factors from the questionnaire survey. Chi-square (χ2) test or Fisher’s exact test will be applied to assess categorical variables on the prevalence of STHs and schistosomiasis. χ2 Test for trend will be used for evaluating ordered categorical variables on the prevalence of STHs and schistosomiasis. The univariate analysis will be carried out to identify the association between potential risk factors and prevalence. I will conduct multivariate logistic regression analysis to determine which risk factors are associated with the outcomes of the prevalence of STHs and schistosomiasis, with the risk factors significantly (p-value < 0.05) associated with hookworm and schistosomiasis infection.

Continuous variables such as eggs counts for the dependent variable will be assessed by using t-test.

When analysing the results of the trial of fatty meal co-administration with single-, and dual-dose albendazole, the primary outcomes will be cure rate and egg reduction rate among four different groups, Chi-square (χ2) test will be applied for analysing the difference of cure rate among four treatment groups. Differences in eggs count between enrolment and follow-up will be assessed by using paired t-test. Chi-square (χ2) test or Fisher’s exact test will be applied to assess categorical variables on the difference of cure rate with four different options of treatment against hookworm. χ2 Test for trend will be used to evaluate ordered categorical variables on the difference of cure rate with four different treatment options against hookworm.

- - **Precautionary measures for the prevalence survey in the context of COVID-19**.

The WHO published the interim guideline of conducting a variety of NTDs related activities in the context of COVID-19 in the name of considerations for implementing mass treatment, active case finding and population-based surveys for neglected tropical diseases in the context of COVID-19 pandemic: interim guidance(43). According to this guideline, for conducting prevalence survey safely during the COVID-19 pandemic situation, there must be following precautionary measures as indicated in the WHO guideline. There are considerations how to take precautionary measures applicable to designated sites for planned activities (table 3 in the interim guidance), precautionary measures for health staff and community health workers (table 4 in the interim guidance), and precautionary measures for the target population(table 5 in the interim guidance).

1. Dissemination Plan

This survey will be conducted with multiple stakeholders including the ministry of health of Uganda, donor organisation (KOICA), scientists from academic partners (London School of Hygiene and Tropical Medicine), policymakers, and health professionals. The results will be written and made academic transcripts for dragging attention to reducing the NTDs burden in Uganda. The results will not reflect any personal information.

Therefore, when the study is complete, the study team will compile and analyse the data, and the overall results will be shared with the study participants and their parents/guardians. The research findings will be disseminated to the Ugandan authorities, donor organisation, policymakers, scientists, and the general public through a variety of forms such as writing, reporting, and presenting in scientific meetings. Only anonymised, summary level data will be included, and your child will not be identifiable from any information shared.

1. Data ownership
2. Collected data will be stored at World Vision ODK server.
3. Any kind of paper based documents will be stored locked cabinets in the World Vision office.
4. The research findings will be disseminated to the Ugandan authorities, donor organisation, policymakers, scientists, and the general public through a variety of forms such as writing, reporting, and presenting in scientific meetings
5. When authorized staff of VCD requests World Vision to share the data, the collected data will be shared with VCD.
6. Timeline

| **Activity** | **February 2021** | **March**  **2021** | **April**  **2021** | **May**  **2021** | **June**  **2021** | **July**  **2021** | **August**  **2021** | **September 2021** | **October**  **2021** | **November**  **2021** | **December**  **2021** | **January**  **2022** | **February**  **2022** |
| --- | --- | --- | --- | --- | --- | --- | --- | --- | --- | --- | --- | --- | --- |
| IRB preparation |  |  |  |  |  |  |  |  |  |  |  |  |  |
| IRB submission  -1) VCD, 2) UNCST |  |  |  |  |  |  |  |  |  |  |  |  |  |
| IRB pass |  |  |  |  |  |  |  |  |  |  |  |  |  |
| Purchase the materials for prevalence survey and the trial |  |  |  |  |  |  |  |  |  |  |  |  |  |
| Prevalence survey and the trial |  |  |  |  |  |  |  |  |  |  |  |  |  |
| Data aggregation, analysis |  |  |  |  |  |  |  |  |  |  |  |  |  |
| Dissemination the result with stakeholders |  |  |  |  |  |  |  |  |  |  |  |  |  |
| Writing report |  |  |  |  |  |  |  |  |  |  |  |  |  |

# Reference

1. Pullan RL, Smith JL, Jasrasaria R, Brooker SJ. Global numbers of infection and disease burden of soil transmitted helminth infections in 2010. Parasites and Vectors. 2014;

2. Brooker S, Clements ACA, Bundy DAP. Global Epidemiology, Ecology and Control of Soil-Transmitted Helminth Infections. Advances in Parasitology. 2006.

3. Bartsch SM, Hotez PJ, Asti L, Zapf KM, Bottazzi ME, Diemert DJ, et al. The Global Economic and Health Burden of Human Hookworm Infection. PLoS Negl Trop Dis. 2016;

4. World Health Organization. Soil-transmitted helminth infection [Internet]. 2019. Available from: https://www.who.int/news-room/fact-sheets/detail/soil-transmitted-helminth-infections

5. Ross, A G.Bartley. P., Sleigh AC, Olds GR, Li Y, Williams GM MD. Schistosomiasis. N Engl J Med. 2002;346(16):1212–20.

6. Gryseels B, Polman K, Clerinx J, Kestens L. Human schistosomiasis. Lancet. 2006;368(9541):1106–18.

7. Ross AGP, Chau TN, Inobaya MT, Olveda RM, Li Y, Harn DA. A new global strategy for the elimination of schistosomiasis. International Journal of Infectious diseases; 2017.

8. Okoyo C, Campbell SJ, Williams K, Simiyu E, Owaga C, Mwandawiro C. Prevalence, intensity and associated risk factors of soil-transmitted helminth and schistosome infections in Kenya: Impact assessment after five rounds of mass drug administration in Kenya. PLoS Negl Trop Dis. 2020;14(10):e0008604.

9. Knopp S, Stothard JR, Rollinson D, Mohammed KA, Khamis IS, Marti H, et al. From morbidity control to transmission control: time to change tactics against helminths on Unguja Island, Zanzibar. Acta Trop. 2013;128(2):412–22.

10. Lo NC, Addiss DG, Hotez PJ, King CH, Stothard JR, Evans DS, et al. A call to strengthen the global strategy against schistosomiasis and soil-transmitted helminthiasis: the time is now. Lancet Infect Dis. 2017;17(2):e64–9.

11. World Health Organization. Preventive chemotherapy to control soil-transmitted helminth infections in at-risk population groups [Internet]. Available from: https://www.who.int/nutrition/publications/guidelines/deworming/en/

12. Crompton DWT. Preventive chemotherapy in human helminthiasis: coordinated use of anthelminthic drugs in control interventions: a manual for health professionals and programme managers. World Health Organization; 2006.

13. Montresor A, Crompton DWT, Hall A, Bundy DA, Savioli L, Organization WH. Guidelines for the evaluation of soil-transmitted helminthiasis and schistosomiasis at community level: a guide for managers of control programmes. World Health Organization; 1998.

14. World Health Organization. Ending the neglect to attain the sustainable development goals. A road map for neglected tropical diseases 2021–2030 [Internet]. 2021. 1–55 p. Available from: https://www.who.int/neglected_diseases/Ending-the-neglect-to-attain-the-SDGs--NTD-Roadmap.pdf

15. Ugandan government launches a national sustainability plan for neglected tropical diseases [Internet]. Act to End NTDs. 2021. Available from: https://www.acteast.org/news/ugandan-government-launches-a-national-sustainability-plan-for-neglected-tropical-diseases

16. Keiser J, Utzinger J, J. K. Efficacy of current drugs against soil-transmitted helminth infections: Systematic review and meta-analysis. JAMA - J Am Med Assoc [Internet]. 2008;299(16):1937–48. Available from: http://jama.ama-assn.org/cgi/reprint/299/16/1937

17. Vercruysse J, Albonico M, Behnke JM, Kotze AC, Prichard RK, McCarthy JS, et al. Is anthelmintic resistance a concern for the control of human soil-transmitted helminths? International Journal for Parasitology: Drugs and Drug Resistance. 2011.

18. Von Samson-Himmelstjerna G, Blackhall WJ, McCarthy JS, Skuce PJ. Single nucleotide polymorphism (SNP) markers for benzimidazole resistance in veterinary nematodes. Parasitology. 2007;134(8):1077.

19. Kaplan RM. Drug resistance in nematodes of veterinary importance: a status report. Trends Parasitol [Internet]. 2004;20(10):477–81. Available from: https://www.sciencedirect.com/science/article/pii/S1471492204002041

20. Schwenkenbecher JM, Albonico M, Bickle Q, Kaplan RM. Characterization of beta-tubulin genes in hookworms and investigation of resistance-associated mutations using real-time PCR. Mol Biochem Parasitol. 2007;

21. Albonico M, Wright V, Bickle Q. Molecular analysis of the β-tubulin gene of human hookworms as a basis for possible benzimidazole resistance on Pemba Island. Mol Biochem Parasitol. 2004;

22. Vercruysse J, Behnke JM, Albonico M, Ame SM, Angebault C, Bethony JM, et al. Assessment of the anthelmintic efficacy of albendazole in school children in seven countries where soil-transmitted helminths are endemic. PLoS Negl Trop Dis. 2011;5(3):e948.

23. Kopp SR, Coleman GT, McCarthy JS, Kotze AC. Application of in vitro anthelmintic sensitivity assays to canine parasitology: detecting resistance to pyrantel in Ancylostoma caninum. Vet Parasitol. 2008;152(3–4):284–93.

24. Kotze AC, Kopp SR. The potential impact of density dependent fecundity on the use of the faecal egg count reduction test for detecting drug resistance in human hookworms. PLoS Negl Trop Dis [Internet]. 2008;2(10):e297. Available from: http://ovidsp.ovid.com/ovidweb.cgi?T=JS&PAGE=reference&D=med7&NEWS=N&AN=18827883

25. Nagy J, Schipper HG, Koopmans RP, Butter JJ, Van Boxtel CJ, Kager PA. Effect of grapefruit juice or cimetidine coadministration on albendazole bioavailability. Am J Trop Med Hyg. 2002;66(3):260–3.

26. Lange H, Eggers R, Bircher J. Increased systemic availability of albendazole when taken with a fatty meal. Eur J Clin Pharmacol. 1988;

27. Ceballos L, Krolewiecki A, Juárez M, Moreno L, Schaer F, Alvarez LI, et al. Assessment of serum pharmacokinetics and urinary excretion of albendazole and its metabolites in human volunteers. PLoS Negl Trop Dis. 2018;12(1):e0005945.

28. Soukhathammavong PA, Sayasone S, Phongluxa K, Xayaseng V, Utzinger J, Vounatsou P, et al. Low efficacy of single-dose albendazole and mebendazole against hookworm and effect on concomitant helminth infection in Lao PDR. PLoS Negl Trop Dis [Internet]. 2012/01/03. 2012 Jan;6(1):e1417. Available from: http://www.plosntds.org/article/fetchObjectAttachment.action?uri=info%3Adoi%2F10.1371%2Fjournal.pntd.0001417&representation=PDF

29. P. S, J. U, Z.-W. D, J.-Y. J, J.-X. C, J. H, et al. Efficacy of single-dose and triple-dose albendazole and mebendazole against soil-transmitted helminths and Taenia spp.: a randomized controlled trial. PLoS One [Internet]. 2011;6(9):e25003. Available from: http://www.plosone.org/article/fetchObjectAttachment.action?uri=info%3Adoi%2F10.1371%2Fjournal.pone.0025003&representation=PDF

30. Adoubryn KD, Kouadio-Yapo CG, Ouhon J, Aka NAD, Bintto F, Assoumou A. Intestinal parasites in children in Biankouma, Ivory Coast (mountaineous western region): efficacy and safety of praziquantel and albendazole. Med Sante Trop. 2012;22(2):170–6.

31. Adegnika AA, Zinsou JF, Issifou S, Ateba-Ngoa U, Kassa RF, Feugap EN, et al. Randomized, controlled, assessor-blind clinical trial to assess the efficacy of single-versus repeated-dose albendazole to treat ascaris lumbricoides, trichuris trichiura, and hookworm infection. Antimicrob Agents Chemother. 2014;58(5):2535–40.

32. Agriculture USD of. Food Data Central [Internet]. [cited 2021 Feb 10]. Available from: https://fdc.nal.usda.gov/fdc-app.html#/food-details/171705/nutrients

33. M. A, P. M, A. M, B. K, V. R, S. P, et al. Comparative study of the quality and efficacy of originator and generic albendazole for mass treatment of soil-transmitted nematode infections in Nepal. Trans R Soc Trop Med Hyg [Internet]. 2007;101(5):454–60. Available from: http://ovidsp.ovid.com/ovidweb.cgi?T=JS&PAGE=reference&D=med6&NEWS=N&AN=17129592

34. Belew S, Getachew M, Suleman S, Mohammed T, Deti H, D’Hondt M, et al. Assessment of efficacy and quality of two albendazole brands commonly used against soil-transmitted helminth infections in school children in Jimma Town, Ethiopia. PLoS Negl Trop Dis. 2015;9(9):e0004057.

35. Jiraanankul V, Aphijirawat W, Mungthin M, Khositnithikul R, Rangsin R, Traub RJ, et al. Incidence and risk factors of hookworm infection in a rural community of central Thailand. Am J Trop Med Hyg. 2011;

36. Gabrie JA, Rueda MM, Canales M, Gyorkos TW, Sanchez AL. School hygiene and deworming are key protective factors for reduced transmission of soil-transmitted helminths among schoolchildren in Honduras. Parasites and Vectors. 2014;

37. Gunawardena GSA, Karunaweera ND, Ismail MM. Effects of climatic, socio-economic and behavioural factors on the transmission of hookworm (Necator americanus) on two low-country plantations in Sri Lanka. Ann Trop Med Parasitol. 2005;

38. Tomono N, Anantaphruti MT, Jongsuksuntigul P, Thongthien P, Leerapan P, Silapharatsamee Y, et al. Risk factors of helminthiases among schoolchildren in southern Thailand. Southeast Asian J Trop Med Public Health. 2003;

39. Ensink JHJ, van der Hoek W, Mukhtar M, Tahir Z, Amerasinghe FP. High risk of hookworm infection among wastewater farmers in Pakistan. Trans R Soc Trop Med Hyg. 2005;

40. Riess H, Clowes P, Kroidl I, Kowuor DO, Nsojo A, Mangu C, et al. Hookworm infection and environmental factors in mbeya region, Tanzania: a cross-sectional, population-based study. PLoS Negl Trop Dis. 2013;7(9):e2408.

41. Olsen A, Samuelsen H, Onyango-Ouma W, Riess H, Clowes P, Kroidl I, et al. A study of risk factors for intestinal helminth infections using epidemiological and anthropological approaches. J Biosoc Sci. 2013;7(9):e2408.

42. Adegnika AA, Zinsou JF, Issifou S, Ateba-Ngoa U, Kassa RF, Feugap EN, et al. Randomized, controlled, assessor-blind clinical trial to assess the efficacy of single-versus repeated-dose albendazole to treat ascaris lumbricoides, trichuris trichiura, and hookworm infection. Antimicrob Agents Chemother [Internet]. 2014;58(5):2535–40. Available from: http://aac.asm.org/content/58/5/2535.full.pdf+html

43. World Health Organization. Considerations for implementing mass treatment, active case-finding and population-based surveys for neglected tropical diseases in the context of the COVID-19 pandemic Interim guidance. WHO/2019-nCoV/neglected_tropical_diseases/2020.1; 2020.

1. *Ascaris lumbricoides* and *Trichuris trichiura* positive cases will be treated with Albendazole even though they are excluded from this study. [↑](#footnote-ref-1)
