# Supplementary material for: The impact of dual- versus single-dosing and fatty food co-administration on albendazole efficacy against hookworm among children in Mayuge district, Uganda: Results from a 2x2 factorial randomised controlled trial
Source: PLoS Negl Trop Dis. 2023 Jul 3;17(7):e0011439. doi: 10.1371/journal.pntd.0011439 (PMC10317238; doi:10.1371/journal.pntd.0011439)
Supplement: S1 Table — (DOCX) [file pntd.0011439.s003.docx]

Table S1: Baseline characteristics by dual-versus single-dose albendazole groups

|  | Single dose albendazole (n=112) | Dual dose albendazole (n=110) |
| --- | --- | --- |
| Male n (%) | 59 (52.7) | 54 (49) |
| Mean age (SD) years | 12.2 (1.7) | 12.3 (1.5) |
| Mean height (SD) cm | 146.6 (13.0) | 145.8 (15.9) |
| Mean weight (SD) kg | 35.6 (6.9) | 36.5 (7.1) |
| HAZ (SD) | -0.53 (2.56) | -0.66 (2.24) |
| BAZ (SD) | -0.86 (1.88) | -0.77 (1.48) |
| Co-infection with schistosomiasis (%) | 17 (15.2) | 27 (24.6) |
| Hookworm infection intensity, n (%) | | |
| Light (1-1,999 epg) | 108 (96.4) | 107 (97.3) |
| Moderate (2,000-3,999 epg) | 2 (1.8) | 2 (1.8) |
| Heavy (≥4,000 epg) | 2 (1.8) | 1 (0.9) |
