# Supplementary material for: The impact of dual- versus single-dosing and fatty food co-administration on albendazole efficacy against hookworm among children in Mayuge district, Uganda: Results from a 2x2 factorial randomised controlled trial
Source: PLoS Negl Trop Dis. 2023 Jul 3;17(7):e0011439. doi: 10.1371/journal.pntd.0011439 (PMC10317238; doi:10.1371/journal.pntd.0011439)
Supplement: S2 Table — (DOCX) [file pntd.0011439.s004.docx]

Table S2: Baseline characteristics by avocado versus no avocado groups

|  | Albendazole without avocado  (n=110) | Albendazole with avocado  (n=112) |
| --- | --- | --- |
| Male n (%) | 57 (50.4) | 56 (49.6) |
| Mean age (SD) years | 12.1 (1.7) | 12.3 (1.6) |
| Mean height (SD) cm | 146.7 (12.6) | 145.7 (16.3) |
| Mean weight (SD) kg | 35.5 (6.6) | 36.7 (7.3) |
| HAZ (SD) | -0.44 (2.49) | -0.74 (2.33) |
| BAZ (SD) | -0.89 (1.90) | -0.74 (1.45) |
| Co-infection with schistosomiasis (%) | 22 (20.0) | 22 (19.6) |
| Hookworm infection intensity, n (%) | | |
| Light (1-1,999 epg) | 107 (97.3) | 108 (96.4) |
| Moderate (2,000-3,999 epg) | 2 (1.8) | 2 (1.8) |
| Heavy (≥4,000 epg) | 1 (0.9) | 2 (1.8) |
