# Supplementary material for: The impact of dual- versus single-dosing and fatty food co-administration on albendazole efficacy against hookworm among children in Mayuge district, Uganda: Results from a 2x2 factorial randomised controlled trial
Source: PLoS Negl Trop Dis. 2023 Jul 3;17(7):e0011439. doi: 10.1371/journal.pntd.0011439 (PMC10317238; doi:10.1371/journal.pntd.0011439)
Supplement: S3 Table — (DOCX) [file pntd.0011439.s005.docx]

Table S3. Estimates of cure rate and ERR for the four trial arms.

|  | Single dose albendazole without avocado (n=60) | Single dose albendazole with avocado (n=52) | Dual dose albendazole without avocado  (n=50) | Dual dose albendazole with avocado  (n=60) |
| --- | --- | --- | --- | --- |
| Cure rate (95%CI) | 81.7 (69.6-90.5) | 86.5 (74.2-94.4) | 98.0 (89.4-99.9) | 95.0 (86.1-98.9) |
| (arithmetic mean)  EPG before treatment | 295 | 420 | 240 | 272 |
| EPG after treatment | 28 | 9 | 1 | 11 |
| Point estimates of ERR (95%CI) (arithmetic mean) | 90.4(57.3-98.9) | 97.8(86.8-99.7) | 99.8(99.2-100) | 95.9(87.1-100) |
