# Supplementary material for: The impact of dual- versus single-dosing and fatty food co-administration on albendazole efficacy against hookworm among children in Mayuge district, Uganda: Results from a 2x2 factorial randomised controlled trial
Source: PLoS Negl Trop Dis. 2023 Jul 3;17(7):e0011439. doi: 10.1371/journal.pntd.0011439 (PMC10317238; doi:10.1371/journal.pntd.0011439)
Supplement: S1 Code — (DOCX) [file pntd.0011439.s007.docx]

deviceid: The individual ID of the mobile phone used by the interviewer.

sub_county: 1-14

school: 1-33

height: cm

weight: kg

sex: 1-male, 2-female

birth_date: year-month-day

age: age in years

WAZ : Weight for age

HAZ: Height for age

BAZ: BMI for age

HH-member: The number of household member who live in the same house

ntd: 1-hookworm only, 8-hookworm and schistosomiasis co-infection

stool_ examination_S. mansoni/slide 1 scr: egg counts of S.mansoni from the first slide before treatment

stool_ examination_S. mansoni/slide 2 scr: egg counts of S.mansoni from the second slide before treatment

average(S.mansoni): average egg count of s.mansoni from the two slides

epg(s.mansoni): average EPG of s.mansoni from the two slides (average(S.mansoni) x 24)

stool_ examination_Hookworm/slide 1 scr: egg counts of hookworm from the first slide before treatment

stool_ examination_Hookworm/slide 2 scr: egg counts of hookworm from the second slide before treatment

average(hookworm): average egg count of hookworm from the two slides before treatment

epg(hookworm): average EPG of hookworm from the two slides before treatment (average(hookworm) x 24)

trial/treatment_option(single/dual with/without avocado 1/4): Four treatment groups; 1- single dose without avocado, 2- single dose with avocado, 3- dual dose without avocado, 4-dual dose with avocado

(FW)stool_ examination_Hookworm/slide 1 fw: egg counts of hookworm from the first slide after treatment

(FW)stool_ examination_Hookworm/slide 2 fw: egg counts of hookworm from the second slide after treatment

(FW)average(hookworm): average egg count of hookworm from the two slides after treatment

Cure(y=0, n=1): 0- cured, 1- not cured

(FW)epg(hookworm): average EPG of hookworm from the two slides after treatment (average(hookworm) x 24)

(FW)stool_ examination_Ascaris/slide 1 fw: egg counts of ascaris from the first slide after treatment

(FW)stool_ examination_Ascaris/slide 2 fw: egg counts of ascaris from the second slide after treatment

(FW)average(Ascaris): average egg count of ascaris from the two slides after treatment

(FW)stool_ examination_T. trichuira/slide 1 fw: egg counts of trichuris from the first slide after treatment

(FW)stool_ examination_T. trichuira/slide 1 fw: egg counts of trichuris from the second slide after treatment

(FW)average(trichuris): average egg count of trichuris from the two slides after treatment

(FW)stool_ examination_Others/slides fw: egg counts of other parasites detected from the first or second slide after treatment
