# Supplementary material for: The impact of dual- versus single-dosing and fatty food co-administration on albendazole efficacy against hookworm among children in Mayuge district, Uganda: Results from a 2x2 factorial randomised controlled trial
Source: PLoS Negl Trop Dis. 2023 Jul 3;17(7):e0011439. doi: 10.1371/journal.pntd.0011439 (PMC10317238; doi:10.1371/journal.pntd.0011439)
Supplement: Attachment — Submitted filename: [31 Mar] Responses to reviewers comments.docx [file pntd.0011439.s008.docx]

**<Methods>**

**Responses to reviewer comments:**

Reviewer #1: Kim and colleagues study the efficacy of a single versus a double dose of albendazole and whether concomitant fatty food improves the activity of albendazole against hookworm infection. The rationale is not very novel but the data confirm earlier findings. The manuscript is fairly well written. My main concern is that the investigators do not follow many points of the required standards for clinical trials (i.e.GCP)

- We thank the reviewer for highlighting the importance of GCP and standards for clinical trials. Summarised below are the measures we undertook and related considerations.
- The participants and their parents were provided with information about study procedures in advance of this study and were informed that they could leave the study at any time. We obtained signed consent forms from guardians/parents and signed assent was also obtained from the participants.
- We obtained local IRB approval from Uganda National Drug Authority, Uganda National Council for Science and Technology, and Vector Control Division in Uganda. We also obtained ethical approval from the LSHTM ethics committee in the UK, and the trial was registered with the Pan African Clinical Trials Registry. We realise that we omitted to include the fact that we had Uganda National Drug Authority (NDA) approval in the original manuscript, and have now added this information.
- Data was anonymised and the assessors analysing stool samples (i.e. trial outcomes) were blinded to which treatment group participants were randomized. Data were originally captured on paper CRFs, which were stored in a locked cabinet in World Vision Mayuge office.
- The Ugandan NDA assigned a designated public health specialist and regulatory affairs officer as a monitor of the clinical trial process.
- The study was insured to cover unexpected adverse events or serious adverse events.
- The fact that Albendazole (single and double dose) is a widely used drug, with minimal side effects and harm from decades of clinical use was also a consideration.

Reviewer #2: Introduction: please add Strongyloides stercoralis to the "dominant STH", as it was finally included in the list.

- We have added *Strongyloides stercoralis* as one of the listed STHs.

-Lines 59-65: I would add considerations on WASH and the issue of re-infections if environmental conditions do not change; this also hampers the reduction of STH prevalence.

- We have added content related to these considerations.

-Line 78-79: [active metabolite of albendazole; albendazole...]. Did the authors mean to put a simple comma between albendazole and albendazole, to explain that the active metabolite is albendazole sulphoxide? It is not immediately clear now.

- Yes, we aimed to explain that the active metabolite is albendazole sulphoxide, and have inserted the comma to make this clear, as suggested by the reviewer.

- I would state in the Methods that the CONSORT checklist was followed.

- We have added this.

-Lines 124-126 (trial exclusion criteria): "to avoid the residual effect of previously administered drugs of albendazole": I would delete "of albendazole". Criterion 2: I would change as "Child who had a history of allergic reaction to any benzimidazole drugs"

- We have amended the text as suggested.

-Trial design, interventions and randomization paragraph, lines 145-146: it is not clear to me why the participants were instructed to have a fatty meal while the investigators wanted to standardize the fat introduction depending on the randomization arm. Were all participants invited to have a fatty meal before reaching the study site? Why?

- The participants were instructed NOT to have a fatty meal but to have a (usual) meal (i.e. not fasted). We have edited the text here to make this point clearer.

-Lines 150-151: where was the randomization list kept? Who kept it?

- The first author (EK) generated the randomisation table and kept it in a locked drawer at the World Vision Mayuge office. On the day of the trial the randomisation list was distributed to the four health centres. The health worker in charge of each health centre then used the randomisation list to assign the participants to one of four treatment groups.

Drug administration and adverse events paragraph. I think that lines 162-164 better fit the Discussion rather than the methods

- We have deleted the contents of lines 162-164 and moved the content described to the Discussion section.
  --------------------

  **<Results>**

  Reviewer #1: Abstract: delete this sentence as it does not fit in this context (the activity observed was not worrisome):  There is growing concern about the low effectiveness of single-dose albendazole, for which there are several possible explanations, including drug resistance and lower absorption using drugs from certain manufacturers.
- Thank you for the suggestion, we have done this.

.
Introduction:
Line 48: I would provide the most recent DALY figures or at least a range

- We have inserted 95% uncertainty interval (UI) as indicated.

Line 56: the albendazole dose for 1-2-year-old children is 200 mg

- We have modified the sentence as indicated.

(400mg of albendazole for adults and over three years old child, 200mg of albendazole for one to two years old child or 500mg of mebendazole)

Line 59:  where are the prevalence still high? I would shift this entire paragraph (line 59-65) after the following paragraph (lines 66-75)

- We have shifted the sentence as suggested.

Line 92-96: was the trial conducted according to GCP? It seems important elements of the GCP practice, as monitoring etc. are missing.

- We thank the reviewer for highlighting the omission on our part. As well as the ethical approvals already described, the Ugandan National Drug Authority (NDA) approval was obtained and is described in the revised manuscript. The Ugandan NDA also approved the designated monitoring expert who monitored the trial and ensured that GCP standards were met. We have now included this information in the ethics section. Please also see the response to Reviewer 1 above summarizing considerations/measures taken to comply with GCP and standards for clinical trials.

Line 113-115: I would delete the design here as it is mentioned below

- We deleted these words as indicated.

Line 145: if the participants had already a meal in the morning prior to the study medication how could you control for a food effect

- Due to the randomized nature of this study, we can assume that different food types are likely to be balanced between trial arms. Furthermore, our study is likely to be representative of “real life” conditions in which people are given MDA. We examined the effect of the reported time interval i.e. time between breakfast and the time at which participants took the trial drug, and found no association between the time interval and cure. We also asked if the participants ate avocado when having breakfast only one participant said "yes" to eating avocado in the morning before coming to the health centre.

Line 162-166: this does not fit here, as already mentioned in the discussion it can be deleted here

- We have deleted the line 162-164 (“… Nagy *et al*. provided a set of Big Mac, French fries, and shake as a fatty meal, which contained 57 grams of fat (12). These amounts of a fatty food, compared to water use, conferred a 6.5-fold increase in the maximum concentration (Cmax) of albendazole sulphoxide, the active metabolite of albendazole…”) as suggested.
- But, we retained lines 165-166 (“For those in the relevant trial arms in our study, we provided 200 grams of avocado, which contains 27-28 grams of fat (29) and is widely available in Uganda at low cost”), since this comprised key information on avocado use as one of the interventions in our study.

Line 169-173: explanation not needed as 14-21 days is the recommended period to take follow up samples.

- We have deleted Line 169-170 (“It takes approximately five to eight weeks for human infected filariform of hookworm to become adult 169 worm and sexually maturate inside the infected human body (30).”) as indicated.

Line 182: how many samples were collected from each participant?

- Each participant submitted one stool sample in the given stool container. We have now clarified this in the text.

Line 206: ODK/Excel should not be used in clinical trials as no audit trail
Results

- For the clinical trial, we recorded the trial data in the paper-based case report forms (CRFs) at first. Then, data in the CRFs was entered on KoBoToolbox for data analysis. The entered data was reviewed and cross-checked alongside the paper CRFs by one of co-authors (DL) before data analysis. When DL identified entry errors, DL informed the primary author(EK) and corrected the data accurately. Therefore, there is a paper-based audit trail.

 Line 256: were

- Thank you for highlighting, we have now corrected this.

Line 263-274: all data is shown in the table and hence this paragraph can be considerably shortened

- We have revised the paragraphs as indicated.

Line 276 line 288 and line 305: efficacy

- We are keeping effectiveness as we explained above (We weighed up the use of effectiveness and efficacy and arrived at the decision to use “effectiveness” due to the real world setting of our randomized trial. While treatment allocation was controlled, we were still subject to the realities of normal operational settings (e.g. what breakfast participants had eaten as highlighted earlier) and we opted to use the word effectiveness throughout. However, we would be happy to re-visit this if the Editor would advise us to use “efficacy” instead.)

Discussion
Line 322: not necessarily, as for intestinal nematodes gut concentrations are more important than plasma levels, so whether a co-administration of fat or an activity against T. trichiura will be observed is questionable

- As pointed out, for treating intestinal nematodes, the active metabolite of albendazole (Albendazole sulphoxide) dose in the gut might be more important than that of plasma level. So we have revised the sentence accordingly to reflect this. Alongside this, we have maintained text describing the possibility that a high dose of the intraluminal active metabolite of albendazole is critical to increasing albendazole effectiveness against hookworm infection. This is because albendazole sulphoxide is an active form to disrupt microtubule formation in parasites.
- We understand that further studies must be followed to verify how the active metabolite of albendazole can remain intraluminal space for a longer period of time.

.

Line 331 and following: can be deleted here, as already mentioned in the limitations below

- We have deleted Line 332-335 (“We used 200 grams of avocado as a fatty food containing 27-28 grams of fat (29), which is half of the amount of fat (57 grams of fat) used in Nagy *et al.’s* study (12). Therefore, we can postulate that 200 grams of avocado may not be adequate to increase albendazole absorption sufficiently to improve albendazole effectiveness against hookworm infection”) as suggested.

Line 341: I would add here that you used a generic product and not the most commonly used GSK product

- We have added a sentence on this - “International pharmaceutical companies such as GSK have donated albendazole, commonly used for MDA in many countries. However, the authors used generic albendazole available in Uganda's domestic market since it is not permitted to use the donated GSK albendazole for clinical trial purposes”

Line 349: preventive chemotherapy is based on a single dose for practicality reason, how would you administer a double dose?

- MDA in Uganda has mainly proceeded in school. Therefore, we consider that dual dose albendazole distribution applying two days’ consecutive provision might be feasible even though a two days’ approach might be more burdensome for teachers and ministry of health supervisors than a single day approach. While we accept dual dosing would be more burdensome, the benefits from dual dosing would make it worthwhile, especially in areas where STH prevalence is still high after a long period of MDA implementation such as Mayuge district, Uganda.

  Line 367/368: delete, this will be part of a data availability statement
- As pointed out, the authors deleted Line 367/368. And we mentioned data availability in the statistical analysis section (Data S1 and Code S1).

The references should be revised (often page numbers etc. missing)
The study protocol is not sufficient for a clinical trial, e.g. study synopsis, investigators, insurance, monitoring etc. are missing
Consort checklist missing

- Thank you for your comments. We believed that the contents of the study synopsis are included in the attached study proposal (protocol S1). The investigators' information is purposely deleted from the submitted protocol S1 since personal and confidential information has been included. However, when we presented the study protocol to the IRBs, the investigators' information was included too.
- We obtained local insurance before conducting the clinical trial. We got the study permission from National Drug Authority because the study team had already received clinical trial insurance from a local insurance company (GoldStar Insurance company) in Uganda.
- We designed, recorded and reported this trial according to the GCP standard. In addition, the designated monitoring expert approved by Ugandan NDA monitored this trial as to whether this trial was conducted according to the GCP standard.
- Consort check list was already attached as supporting information (Checklist S1).
- We have revised References.

  Reviewer #2: Table 1 - I would report the acronyms used in the table as footnotes. HAZ is missing also from line 266
- We explain the acronyms in the text and have now added footnotes to Tables as suggested.

Effectiveness of results: actually in a clinical trial I would talk about efficacy rather than effectiveness. Main results should be reported either in the Tables or in the text, otherwise are redundant. Tables 2 and 3: I think that adding a column reporting OR and p values would be better than reporting them in a line, unrelated to the values they are comparing.

- Thank you for your comments. We weighed up the use of effectiveness and efficacy and arrived at the decision to use “effectiveness” due to the real world setting of our randomized trial. While treatment allocation was controlled, we were still subject to the realities of normal operational settings (e.g. what breakfast participants had eaten as highlighted earlier) and we opted to use the word effectiveness throughout. However, we would be happy to re-visit this if the Editor would advise us to use “efficacy” instead. .

  --------------------

  **<Conclusion>**

  Reviewer #1: (No Response)

  Reviewer #2: The conclusions are supported by the results. I would add some further considerations:
  - For increasing effectiveness and reducing emergence of resistance, combination of benzimidazole drugs with ivermectin is also considered. This combination is already present in the WHO model list
- Thank you for your comment. We have inserted this phrase in the conclusion.

-For MDA, it is difficult to administer more than a single day regimen in many settings. Also in light of this, drug combination would have advantages compared to increasing doses of albendazole/mebendazole

- Thank you for your comment. We have inserted this phrase in the conclusion.

Line 369: from here on, the Authors draw the main conclusions. I would hence add the "conclusions" title.

- Thank you for your kind comment. We added the “conclusion” title.

  --------------------

  **<Editorial and Data Presentation Modifications>**

  Reviewer #1: (No Response)

  Reviewer #2: (No Response)

  --------------------

  **<Summary and General Comments>**

  Reviewer #1: (No Response)

  Reviewer #2: The relevance of this study is mainly on the fatty meal component, as the reduced efficacy of a single administration of albendazole, in specific settings, is quite known. The trial was well conducted and the results are reported clearly, although some revisions should be done in order to make the text less redundant (considering the Tables). Although the results here point out at an increased efficacy of multiple doses of albendazole, in the Discussion I would point out that giving multiple doses in a MDA context is not always feasible, hence other strategies should hence be explored (such as combination with fatty meal and drug combinations)
- Thank you for your kind comment. We agree with the idea of a drug combination (albendazole with ivermectin) as an alternative recommendation for improving the drug's effectiveness. On the other hand, we still consider using dual or triple-dose albendazole could be a feasible and reasonable option in a real-life setting because it might be challenging to find ivermectin in the local market, and the ivermectin price is higher than albendazole.

  --------------------

  **<Do you want your identity to be public for this peer review?>**

  Reviewer #1: No

  Reviewer #2: No
