# Supplementary material for: The impact of dual- versus single-dosing and fatty food co-administration on albendazole efficacy against hookworm among children in Mayuge district, Uganda: Results from a 2x2 factorial randomised controlled trial
Source: PLoS Negl Trop Dis. 2023 Jul 3;17(7):e0011439. doi: 10.1371/journal.pntd.0011439 (PMC10317238; doi:10.1371/journal.pntd.0011439)
Supplement: Attachment — Submitted filename: [May 2023] responses to reviewers comments.docx [file pntd.0011439.s009.docx]

**Methods**

Reviewer #1: The authors have done a good job revising the manuscript. I would still suggest using the term efficacy, as often trial data is reported and not as the authors point out, real world scenario (e.g. line 94 introduction). The authors added as much as possibility on the GCP compliance though there are several small issues (e.g. the use of Excel) which should be fixed in future trials. I would also rewrite the argumentation the absorption/fat hypothesis as summarized below.

- 1) We thank you for the reviewer’s sincere consideration and we have accepted the reviewer #1’s suggestion and replace effectiveness to efficacy accordingly throughout the manuscript. We have also replaced “efficacy” by “effectiveness” in the manuscript title.
- 2) Thank you for your kind comment on the GCP compliance. We will reflect the reviewer #1’s suggestion and apply the reviewer #1’s opinion in future trials.

**Abstract**
Could be merged with previous sentence: The difference of ERR between the two groups (with and without avocado) was 2.8% (95% CI -8.63-14.3%, p=0.629).

- 1) We merged two sentences into one sentence as below;

The ERR was 97.0% and 94.2% in the group receiving albendazole with and without avocado, respectively, and the difference in ERR between the two groups was 2.8% (95% CI -8.63-14.3%, p=0.629).

**Author summary**
Last sentence, I am not convinced as it might not require an improved absorption but rather dissolution

- Thank you for your comment. After carefully considering your advice, we have revised the expression from "Improved absorption with high fat levels" to "improving albendazole efficacy". While we did not find in our study that co-administration with a high-fat food improved albendazole efficacy against hookworm infection, we think that further exploration to identify biological rationales (which could include improved absorption but also improved dissolution) may be necessary to understand how to increase albendazole efficacy for the treatment of intraluminal helminth infections.

**Introduction**
Line 89: albendazole

Line 112: council

- Thank you for pointing out the misspelling. We’ve corrected those words.

**Sample size**
Line 212: the cure rate assumption 50% versus 70% is very optimistic—a single dose of albendazole has quite a good efficacy and I am surprised by the 54% value. At the end the difference was around 10%-was the study underpowered? This is not mentioned in the limitation section. What were the assumptions for the avocado arms?

- Thank you for your valuable feedback. We appreciate your attention to detail and would like to acknowledge the typo in the assumed cure rate for single and dual-dose albendazole. Based on our literature review before conducting the clinical trial, we had assumed a cure rate of 80% for single-dose albendazole and 95% for dual-dose albendazole against hookworm infection when determining the sample size for this trial.

According to our literature review, the cure rate of single-dose albendazole on hookworm infection ranges between 35% and 100%. However, Moser et al.(Efficacy of recommended drugs against soil transmitted helminths: Systematic review and network meta-analysis. BMJ 2017;358: j4307) estimated single-dose albendazole drug efficacy was 79.5% in their systematic review. On the other hand, the data regarding the cure rate of dual-dose albendazole on hookworm infection is limited except the study by Adegnika et al.’s Gabon study (Adegnika et al. Randomized, controlled, assessor-blind clinical trial to assess the efficacy of single-versus repeated-dose albendazole to treat ascaris lumbricoides, trichuris trichiura, and hookworm infection. Antimicrob Agents Chemother. 2014;58(5):2535–40). Adegnika et al. compared the cure rate of single-dose, dual-dose, and triple-dose albendazole on hookworm infection. The cure rate was 54%, 92%, and 93%, respectively.

Studies reported cure rate of triple-dose albendazole on hookworm infection to be between 92% and 98%, within the limited reference on dual-dose and triple-dose albendazole drug efficacy. We assume that the cure rate of dual-dose albendazole on hookworm infection is similar to that of triple-dose albendazole according to Adegnika et al.’s study conducted in Gabon.

We have created the table below showing various scenarios applying different cure rates of single-dose (50-90%) and dual-dose albendazole (70-95%) on hookworm infection. Of different scenarios of cure rates, based on the Moser et al. systematic review, We assume that the cure rate of single-dose albendazole on hookworm infection is 80%, and the cure rate of dual-dose albendazole on hookworm infection is 95%. With this assumption, applying type I error of 5%, power of 90%, and 5% follow-up loss three weeks after the trial by albendazole treatment, the total required sample size would be 210. This scenario is highlighted in bright yellow; other scenarios for which the trial will have specified power are highlighted in pale yellow.

| Cure rate | | Power 80% (*) | | Power 90% (**) | |
| --- | --- | --- | --- | --- | --- |
| Single-dose | Double-dose | Sample size | Sample size (5% follow up loss) | Sample size | Sample size (5% follow up loss) |
| 50 | 70 | 186 | 195.3 | 248 | 260 |
| 50 | 80 | 76 | 79.8 | 102 | 107 |
| 50 | 90 | 38 | 39.9 | 50 | 53 |
| 50 | 95 | 28 | 29.4 | 38 | 40 |
| 60 | 70 | 712 | 747.6 | 952 | 1000 |
| 60 | 80 | 162 | 170.1 | 216 | 227 |
| 60 | 90 | 62 | 65.1 | 84 | 89 |
| 60 | 95 | 42 | 44.1 | 56 | 59 |
| 70 | 80 | 586 | 615.3 | 784 | 824 |
| 70 | 90 | 124 | 130.2 | 164 | 173 |
| 70 | 95 | 46 | 70 | 92 | 97 |
| 80 | 90 | 398 | 417.9 | 532 | 559 |
| **80** | **95** | **150** | **157.5** | **200** | **210** |
| 90 | 95 | 868 | 911.4 | 1162 | 1220 |

Regarding the avocado arms, as this was the first clinical trial of its kind, we had to make some arbitrary assumptions regarding the cure rate for these arms, and used the same assumptions as for the dual versus single-dose comparison, i.e. an 80% cure rate for the pure albendazole use group and a 95% cure rate for the albendazole with avocado group.

We have now updated the text to accurately reflect the sample size calculations and give more justification of the assumptions.

**Discussion**
Line 355: why would it not be permitted to use the GSK drug? In worst case it could have been purchased. I would delete this here.

- We tried to find GSK manufacturing albendazole in Uganda's local market. Unfortunately, only GSK-donated albendazoles were available, which the Uganda Ministry of Health (MOH) holds. But MOH didn't allow us to use these GSK drugs since these GSK drugs must be used for MDA, not for clinical trials. So, we had to buy available albendazole in Uganda's local market for the clinical trial.

Line 357-360 it is not really clear how this argument relates to the different brands of tablets. Would this not rather belong to the discussion on the fatty food?

- Belew et al., in their Ethiopian study, compared the albendazole efficacy with two different brands of albendazole, calculating ERR and showed the dissolution rates of the two other drugs simultaneously. For example, Ovis, produced by Daehwa company, has a higher dissolution rate than Bendex, produced by CIPLA company. And the ERR against hookworm is 98.1% and 88.7%, respectively. So, the argument in lines 357-360 can be related to the different brands of tablets, and was included here to highlight the fact that we used the same brand and batch of albendazole for treating all trial participants, and so differences between brands could not influence our results.

Line 379: is it really worth to conduct further studies against Trichuris and with a higher fat dose? At the end it is pure speculation and there is no evidence why the drug should perform better in the presence of fat. We do not know what is driving efficacy, e.g. intrgastrical concentrations or plasma levels.

- We are slightly unclear on which line the reviewer refers to as we do not mention Trichuris. However, assuming that this comment is on our suggestion that further work should be done to investigate the impact of co-administration with fatty foods, we note the following. According to the finding we identified from the clinical trial, there is no evidence of improving the drug efficacy against hookworm when using fat-containing food. However, absence of evidence is not the same as evidence of absence, and given the preliminary data from Nagy et al, there is still a rationale for investigating how to enhance albendazole efficacy by co-using supplementary foods, including those with high fat content. Avocado is a food containing high fat and, at the same time, it contains high fibre. Improving albendazole absorption might not cause a corresponding increase inalbendazole efficacy against intraluminal hookworm infection. But, other hypotheses might be possible for the future trial. For example, foods increasing gut-transit time could improve albendazole efficacy against intraluminal helminth infection. And for extraintestinal helminths, the hypothesis of high amounts of fatty food could still be valuable to increase albendazole absorption. Therefore we have revised line 379 as below.

**“The 27-28 grams of fat contained in 200 grams of avocado in our study, may be too low in fat. Further work is needed to determine whether higher amounts of fat could increase efficacy of albendazole”.**

Similar on line 399—there is no correlation with regard to systemic availability and efficacy from PK/PD studies, so I would challenge your last sentence that due to the biological rationale for improved absorption with high fat levels.

- Although we don’t prove the benefit of co-administration of avocado to treat intraluminal hookworm, further studies would still be valuable to prove the effectiveness of co-administrating with albendazole for treating intraluminal helminth infection. So, we changed the last sentence as below:

**“While we did not demonstrate any benefit with use of avocado co-administration, there remains scope for future studies to investigate whether albendazole efficacy against intraluminal helminth infection could be improved through co-administration with different types of food.”**

Reviewer #2: (No Response)

--------------------

**Results**


Reviewer #1: trial raw data should be provided (egg counts etc)

- We have already submitted the raw data (Data S1). Column Y in the data S1 shows the hookworm egg counts(eggs per gram, epg) before treating the trial participants with trial drugs, and column AY shows the hookworm egg counts(epg) after treatment.

  Reviewer #2: The only point that I raised in my previous review and was not addressed concerned the format of Tables 2 and 3: I suggested to add a column reporting the results of the statistical tests (OR and p) for clarity. I still believe that would be better than having OR and p in a line
- Thank you for your comment. We revised tables 2 and 3, creating separate columns showing OR and p-value in a line as you instructed. We have also included the difference in ERR (95% CI and p-value) in this column.

  --------------------

  **Conclusions**

  Reviewer #1: (No Response)

  Reviewer #2: (No Response)

  --------------------

  **Editorial and Data Presentation Modifications?**

  Reviewer #1: (No Response)

  Reviewer #2: (No Response)

  --------------------

  **Summary and General Comments**

  Reviewer #1: (No Response)

  Reviewer #2: I am satisfied by the authors' reply and revisions. As I pointed out previously, the only point that I still have concerns the Tables 2 and 3.
- As mentioned above, we revised the tables 2 and 3 as you instructed. Thanks again.

  --------------------

  Do you want your identity to be public for this peer review?

  Reviewer #1: No

  Reviewer #2: No
